# A framework for deformable image registration validation in radiotherapy clinical applications

**DOI:** 10.1120/jacmp.v14i1.4066

**Published:** 2013-01-02

**Authors:** Raj Varadhan, Grigorios Karangelis, Karthik Krishnan, Susanta Hui

**Affiliations:** ^1^ Minneapolis Radiation Oncology Minneapolis MN USA; ^2^ Oncology Systems Limited Shrewsbury Shropshire UK; ^3^ Kitware, Inc. Clifton Park NY USA; ^4^ Department of Therapeutic Radiology University of Minnesota Minneapolis MN USA

**Keywords:** deformable image registration, deformation vector field, inverse consistency error, quality assurance, adaptive radiotherapy, image‐guided radiation therapy

## Abstract

Quantitative validation of deformable image registration (DIR) algorithms is extremely difficult because of the complexity involved in constructing a deformable phantom that can duplicate various clinical scenarios. The purpose of this study is to describe a framework to test the accuracy of DIR based on computational modeling and evaluating using inverse consistency and other methods. Three clinically relevant organ deformations were created in prostate (distended rectum and rectal gas), head and neck (large neck flexion), and lung (inhale and exhale lung volumes with variable contrast enhancement) study sets. DIR was performed using both B‐spline and diffeomorphic demons algorithms in the forward and inverse direction. A compositive accumulation of forward and inverse deformation vector fields was done to quantify the inverse consistency error (ICE). The anatomical correspondence of tumor and organs at risk was quantified by comparing the original RT structures with those obtained after DIR. Further, the physical characteristics of the deformation field, namely the Jacobian and harmonic energy, were computed to quantify the preservation of image topology and regularity of spatial transformation obtained in DIR. The ICE was comparable in prostate case but the B‐spline algorithm had significantly better anatomical correspondence for rectum and prostate than diffeomorphic demons algorithm. The ICE was 6.5 mm for demons algorithm for head and neck case when compared to 0.7 mm for B‐spline. Since the induced neck flexion was large, the average Dice similarity coefficient between both algorithms was only 0.87, 0.52, 0.81, and 0.67 for tumor, cord, parotids, and mandible, respectively. The B‐spline algorithm accurately estimated deformations between images with variable contrast in our lung study, while diffeomorphic demons algorithm led to gross errors on structures affected by contrast variation. The proposed framework offers the application of known deformations on any image datasets, to evaluate the overall accuracy and limitations of a DIR algorithm used in radiation oncology. The evaluation based on anatomical correspondence, physical characteristics of deformation field, and image characteristics can facilitate DIR verification with the ultimate goal of implementing adaptive radiotherapy. The suitability of application of a particular evaluation metric in validating DIR is dependent on the clinical deformation observed.

PACS numbers: 87.57 nj, 87.55‐x,87.55 Qr

## I. INTRODUCTION

Image‐guided radiation therapy (IGRT) has become a widely used treatment modality in recent past with advanced treatment processes. IGRT requires daily or frequent imaging, which can lead to treatment planning modification decisions based on patient‐specific anatomical variations as quantified by the imaging. However, routine IGRT in most clinical departments uses only the vendor‐supplied rigid registration matching between original treatment planning CT (kvCT) and the daily imaging study set.

Deformable image registration (DIR) studies have been advocated to more accurately quantify these anatomical and biological variations.^(^ ^1^ ^)^ Deformable registration is essential to map the position of each voxel to a reference CT image for dose tracking and to ultimately practice adaptive radiotherapy.^(^ ^2^ ^,^ ^3^ ^)^ The accuracy of deformable registration is particularly important in intensity‐modulated radiation therapy (IMRT) and adaptive radiotherapy that deliver differential doses to different parts of the tumor and organs at risk, which then sum to a uniform dose. In addition, DIR has important applications in other categories of image‐guided medical interventions, as well.^(^ ^4^ ^–^ ^10^ ^)^ The existing methods of deformable image registration can be classified broadly into two categories: parametric or model‐based (such as B‐spline,^(^ ^11^ ^)^ thin‐plate spline,^(^ ^12^ ^)^ and linear elastic finite element^(^ ^13^ ^)^, and nonparametric methods (e.g., optical flow,^(^ ^14^ ^)^ viscous fluid^(^ ^15^ ^)^.

There have been many techniques proposed to validate the accuracy of various DIR algorithms.^(^ ^16^ ^–^ ^24^ ^)^ All DIR evaluation procedures require the use of evaluation data and validation methods. Considering the evaluation data, one can separate the methods into two groups: a) those using real patient image data, and b) those using phantom image data. In the first, the authors use real patient images that they deform artificially to create the reference and the test study. Alternately, multiple imaging acquisitions on different time moments where changes in anatomy are clearly visible and anticipated (e.g., replanning scans or cone‐beam CT scans) are used. The use of deformable phantoms has also been explored to validate the accuracy of DIR. However, phantoms as described in the studies by Serban et al., Kashani et al., and Kirby et al.^(^ ^25^ ^–^ ^27^ ^)^ cannot be routinely used in most busy clinical departments because of the lack of resources and time required to build and test these phantoms. Further, it is not practical to build a phantom that will be sophisticated enough to simulate all anatomical deformations that can occur in a clinical environment. It has also been suggested that the presence of uniform intensity regions in the phantom images, as opposed to more intensity gradients in clinical CT images, may limit the applicability of phantom tests in DIR verification.^(^ ^16^ ^)^

The validation methods often include using landmark points in regions of interest as a surrogate tool in verifying accuracy of DIR. A frequent problem with this technique is locating the landmark points, which in a real patient's anatomy can be time‐consuming and difficult to identify markers in low‐contrast regions. The contour‐based evaluation is useful qualitative verification in contour propagation and also for inspecting anatomical difference among images. Although contour propagation techniques seem to provide a more efficient way of validation compared to markers, including changes in shape volume and location of a structure, they often do not confirm that the volume within the contour has been properly registered.

In this work, we describe a commercial software tool kit, ImSimQA (Oncology Systems Limited, Shrewsbury, Shropshire, UK) which can serve as a virtual deformable quality assurance (QA) tool by simulating clinically observed organ deformations in routine IGRT. In contrast to previous years where deformable registration algorithms where available only in a research based setting, today several commercially available products are available. Most of these commercially available products are “black boxes”, in that very little information is known to the medical physicist regarding the overall system accuracy of the implemented algorithm and what the limitations of the deformable registration algorithm could be for a given clinical situation. This is particularly true for IGRT, since different organs exhibit varying levels of deformation over the course of radiation therapy. Presumably the algorithm will have different registration settings to accurately register the images over these varying clinical scenarios. Therefore, it is critical that some quantitative validation of the system accuracy of the implemented algorithm and its potential limitations in the commonly encountered IGRT clinical situations exists.^(^ ^28^ ^)^

This work describes a complete set of metrics and tools and a practical framework to evaluate a deformation field that will facilitate introducing a particular DIR algorithm to clinical use. The proposed framework also highlights the importance of selecting an appropriate evaluation metric which is dependent on a given clinical deformation. This will ensure that a false positive conclusion is not reached in validating a particular DIR algorithm.

## II. MATERIALS AND METHODS

**Figure 1:**
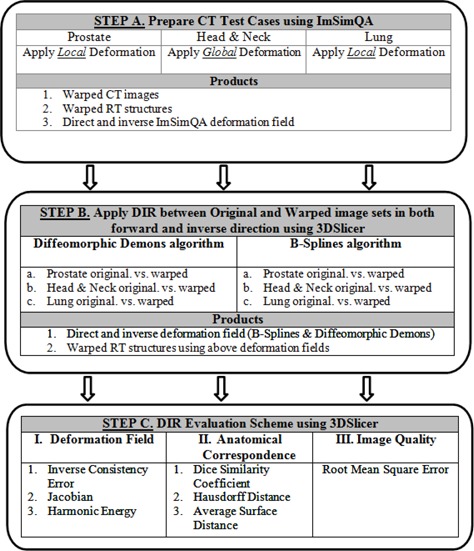
DIR algorithm analysis workflow.

The workflow and evaluation methods for DIR accuracy used in this paper are summarized in the flow chart in Fig. 1.

### A. Deformation process

A brief description of the deformation process used in ImSimQA to warp the images to produce clinically observed organ deformation is presented below. In ImSimQA, there are two choices for the geometric deformation of the image data. Both implementations are based on the radial basis function approach with different kernel functions. For the global deformation of the data, the thin‐plate spline (TPS)^(^ ^29^ ^)^ kernel was utilized and the compact support radial basis functions (CSRBF)^(^ ^30^ ^)^ as the local deformation scheme.

#### A.1 Global deformations

In ImSimQA, the algebraic solution of Bookstein^(^ ^29^ ^)^ is followed which treats the TPS solution as an interpolation problem. In order to perform the deformation, two sets of landmark points must be chosen which will be referred to as the source points (SP) and the target points (TP) from so on. In case of a 2D image, the surface of the image is treated as a 2D grid with each *x, y* of the image coordinates being a part of the image grid. The SP and TP are manually inserted on the grid. The vectors, with their origin at the SP coordinates and directed at the corresponding TP coordinates, show the deformation direction of the grid. In order to solve this problem, a mapping function f(x,y) is found that will map the SPs to TPs by deforming the underlying grid.

#### A.2 Local deformations

The TPS deformation affects the whole image and is characterized as a global deformation procedure. For applications which need local deformation, the CSRBF model is implemented. The locality effect of the CSRBF is adjusted by calibrating a scaling parameter. The algebraic solution to the CSRBF is the same as the TPS with the only difference being the RBF kernel used. The CSRBF kernel is a Wendland function^(^ ^31^ ^)^ constructed from piecewise polynomials. In both algorithms, anchor markers can be placed inside the dataset in places where the restriction of the deformation is needed. If the SP and the TP are identical, then deformation around these markers is restricted. By default, in both algorithms there are anchor points at the border of the dataset, four in the 2D case and eight in the 3D case. This is done to avoid excessive warping of the original dataset.

#### A.3 DIR verification workflow in ImSimQA

ImSimQA can be used as a virtual quality assurance tool by mimicking a clinically observed organ deformation on the treatment planning dataset which can then be used to verify the accuracy of DIR algorithm. For more complex deformations, one can combine TPS over CSRBF deformations and vice versa. The final deformation field is composed as the addition of both deformations which can be applied only once on the original image set.

The new deformed dataset, including deformed radiotherapy (RT) structures and the deformation field as a 3D binary grid, can then be used to compare with the results of DIR algorithm and to facilitate DIR verification.

The deformation algorithms in ImSimQA are controlled using marker points that are user‐defined and can be freely positioned on the image set. The user defines the source control marker points and enables the local (CSRBF) or global (TPS) deformation procedure. The direction of the deformation is given by translating and rotating the control points in three dimensions individually or as a group of points. For the deformation procedure to start, the source and target positions of the control points are used. A deformation field comprising a three‐component vector value at each voxel is then generated. The source image is then warped using this deformation field. Trilinear interpolation is used to correct floating voxel locations during image warping.

**Figure 2:**
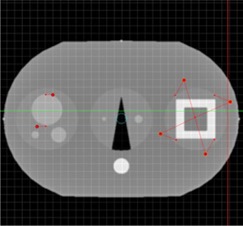
Axial image of quasar phantom with original and target marker points.

**Figure 3:**
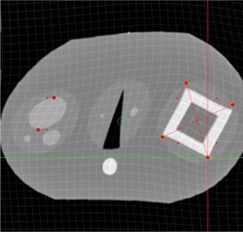
Axial phantom image after applying the global (TPS) deformation algorithm.

**Figure 4:**
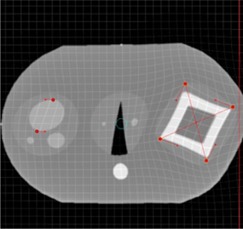
Axial phantom image after applying the local (CSRBF) deformation algorithm.

In Figs. 2 to 4, a simulated deformation example is illustrated. Figure 2 shows the axial image of quasar phantom^(^ ^32^ ^)^ with original and target marker points. The global deformation (TPS) result is shown in Fig. 3. Figure 4 shows the result of local deformation (CSRBF). For both deformations the same source and target points were used.

### B. Clinical rationale and description of applied known deformation in each anatomical site

***Prostate:*** In prostate IGRT, deformation of the prostate due to variations in rectal filling is commonly observed. The need for adaptive radiation therapy for prostate cancer due to inter‐ and intrafraction motion is well documented in the literature.^(^ ^33^ ^–^ ^43^ ^)^ We applied a known deformation in the ImSimQA to mimic a distended rectum and introduce rectal gas in the synthetically deformed image. This, in turn, deforms the prostate, as routinely seen in prostate IGRT.

The applied deformation was achieved using the following general workflow. Two series of datasets (say A & B) were imported into ImSimQA. Series A is the image set to be deformed and series B is the anatomy we want to match which contains the desired deformation. Anatomical changes that occur in different organs were noted by using the rigid image fusion/blending tool. The first step involved placement of markers on the anatomy which is to be deformed (Series A). The second step involved moving the markers to desired locations by following the borders and internal tissues of the deformed anatomy (Series B). Thus the markers were moved accordingly and in approximation to the deformations that need to be achieved. In the prostate case, this was achieved by tracking the motion of the rectal wall and prostate. This was a trial‐and‐error procedure that had to be repeated iteratively until one could accurately approximate the desired deformations by using the Series B dataset as objective criteria for validation. The gas pocket in the rectum was induced by drawing a set of contours on Series A and then subtracting the Hounsfeld units (HU) from the existing DICOM images. This resulted in the creation of a low‐density object without eliminating the underlying tissue structure. The advantage of using the synthetically deformed series A as opposed to series B for DIR verification is that one now has the deformation vector field (DVF) used to simulate the necessary deformation which will be used to warp the RT structures and can be compared with the DVF arising from DIR algorithms.

**Figure 5:**
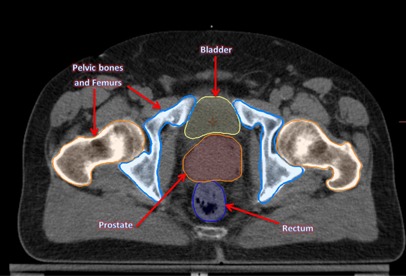
Axial view of the prostate kvCT image with original RT structures: bladder, prostate, rectum, and pelvic bones.

**Figure 6:**
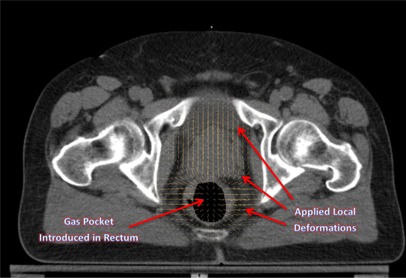
Axial view illustrating the local deformations introduced in the prostate and rectal region, and gas pocket in the rectum.

**Figure 7:**
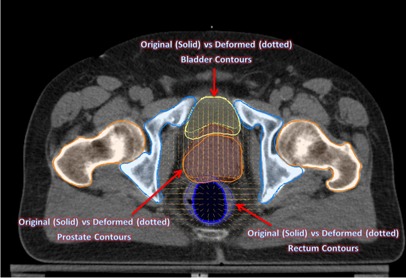
Contour changes in prostate, bladder, and rectum from the applied ImSim DVF when compared with original segmentation of these structures. Solid figures refer to original segmentation done by radiation oncologist on kvCT image and dotted figures refer to the deformed volumes due to the applied deformation.

For the CT series of the prostate case (512×512×74 (median) voxels; $0.86\times 0.86\times 5.0\;{\text{mm}}^{3}$), images were acquired on a SOMATOM Sensation 16 CT scanner (Siemens Medical Solutions, Malvern, PA) which included the RT structures used during DIR evaluation. It should be noted all the applied deformation in this study is fully three‐dimensional, although only a particular slice view is shown for illustration. Figure 5 shows axial view of original kvCT image with RT structures, Fig. 6 indicates applied deformation from ImSimQA, and Fig. 7 shows the changes in RT structures from the applied ImSim DVF when compared to original RT structures.

***Head & Neck:*** There are significant changes in patient anatomy during the course of head and neck IGRT treatment that are related to decrease of tumor and nodal volumes, patient weight, and alteration in muscle and fat distribution, with an average tumor volume reduction of 70% of its initial volume at the end of treatment.^(^ ^44^ ^)^ Similarly, the parotid glands also undergo significant volume reduction with an average reduction of 49.8% and a translation of 8.1 mm upon completion of treatment.^(^ ^45^ ^)^

The changes occurring due to patient weight loss could have a significant impact on organs at risk (OAR) like the parotids, since these structures can now be in the high‐dose gradient area and tumor could be under dosed. Adaptive radiotherapy has been advocated to mitigate such volume changes.^(^ ^46^ ^–^ ^51^ ^)^ We applied a global deformation on the ImSimQA software to change the patient neck flexion, and studied the deformable registration algorithm to track these changes. The desired deformation was achieved in principle using the same workflow as described before, but the TPS algorithm was used for global deformation of the dataset. The anchor and moving markers were iteratively adjusted until a large neck flexion deformation was achieved using the TPS algorithm. The induced deformation does not correspond to interfraction variation that occurs during routine head and neck IGRT, but rather relates to a clinical scenario where the patient was treated previously with a completely different neck position and is now being evaluated for radiation therapy in the same area in a different treatment position. The induced deformation significantly altered the nasal cavity, the alignment of vertebral body, spinal cord, and skull in comparison with the original image.

**Figure 8:**
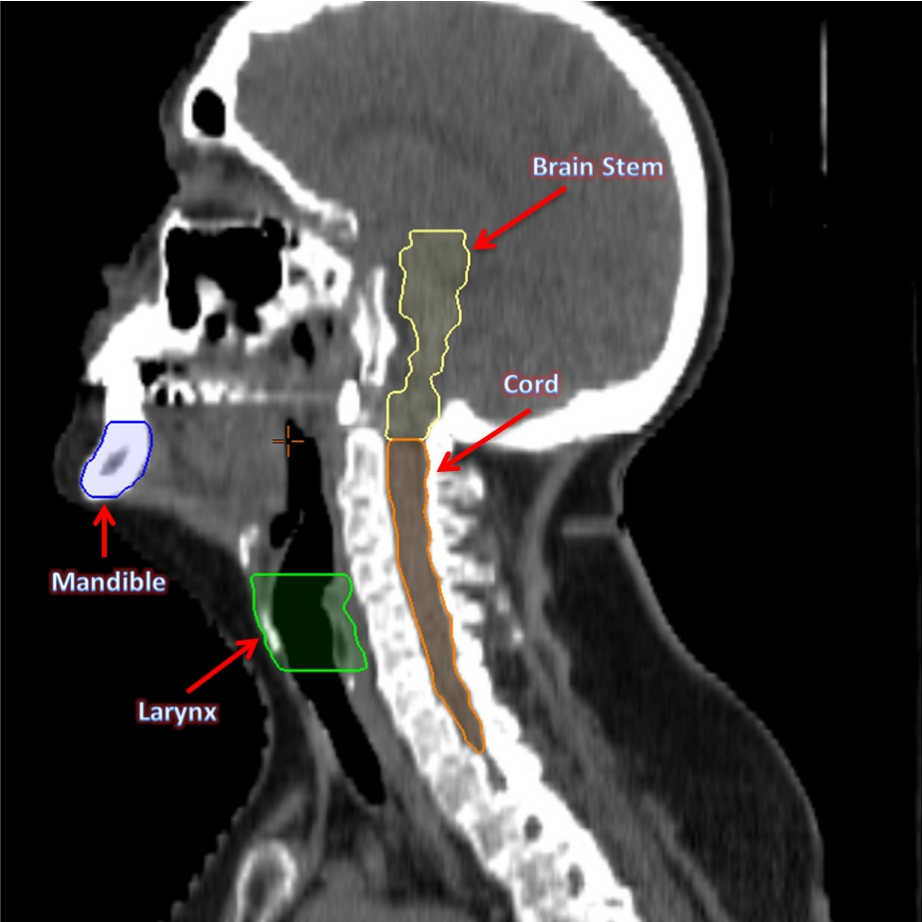
Sagittal view of the original head and neck CT image with the associated RT structures brain stem, cord, larynx, and mandible.

**Figure 9:**
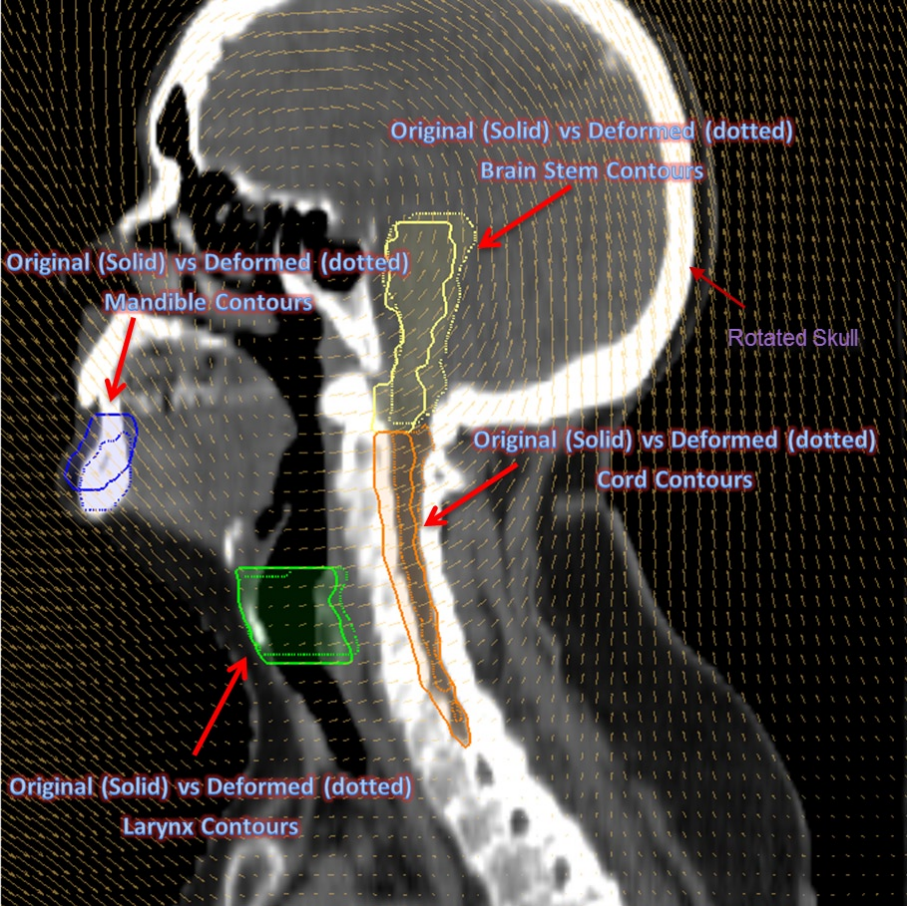
Sagittal view of the head and neck image from the applied global deformation mimicking a large neck flexion. The skull is rotated counterclockwise. The deformed RT structures due to the applied deformation are displayed as dotted figures.

For the CT series of the head and neck case (512×512×112 (median) voxels; $0.94\times 0.94\times 3.0\;{\text{mm}}^{3}$), images were acquired on a SIEMENS Sensation 16 CT scanner (Siemens Medical Solutions) which included the complete set of RT planning structures. Figure 8 shows the sagittal view of original kvCT image with associated RT structures. Figure 9 shows the deformed image from ImSimQA after applying the neck flexion and the warped RT structures as result of applied ImSim DVF when compared to original RT structures.

***Lung:*** Respiratory motion of the order of 1 cm has been observed for tumors close to the diaphragm.^(^ ^52^ ^,^ ^53^ ^)^ We introduced a deformation in ImSimQA to mimic dataset from inhale and exhale breathing phases of 4D CT. This was achieved by picking an exhale and inhale dataset from a patient. Using rigid image fusion tool as guide, anchor and moving markers were placed by following the motion of diaphragm and edges of lung tissue. This process was iteratively repeated until CSRBF algorithm approximated the deformation of the inhale dataset, which was used as the objective criteria for validation of deformation.

In addition to the lung volume changes, we introduced contrast changes in the image to assess the quality of DIR during variable contrast enhancement. The original kvCT images have contrast in the scan, while in the synthetically deformed images from ImSimQA, the contrast has been taken out without eliminating the underlying tissue structure. This was done by contouring the contrast in the deformed dataset and subtracting the HU values from the DICOM dataset. This example was chosen to highlight the limitations of diffeomorphic demons algorithm when the intensities of identical tissues and organs are different in the two images.

For the CT series of the lung case (512×512×123 (median) voxels; $0.98\times 0.98\times 3.0\;{\text{mm}}^{3}$), images were acquired on a SOMATOM Sensation 64 unit (Siemens Medical Solutions), which included the complete set of RT planning structures.

**Figure 10:**
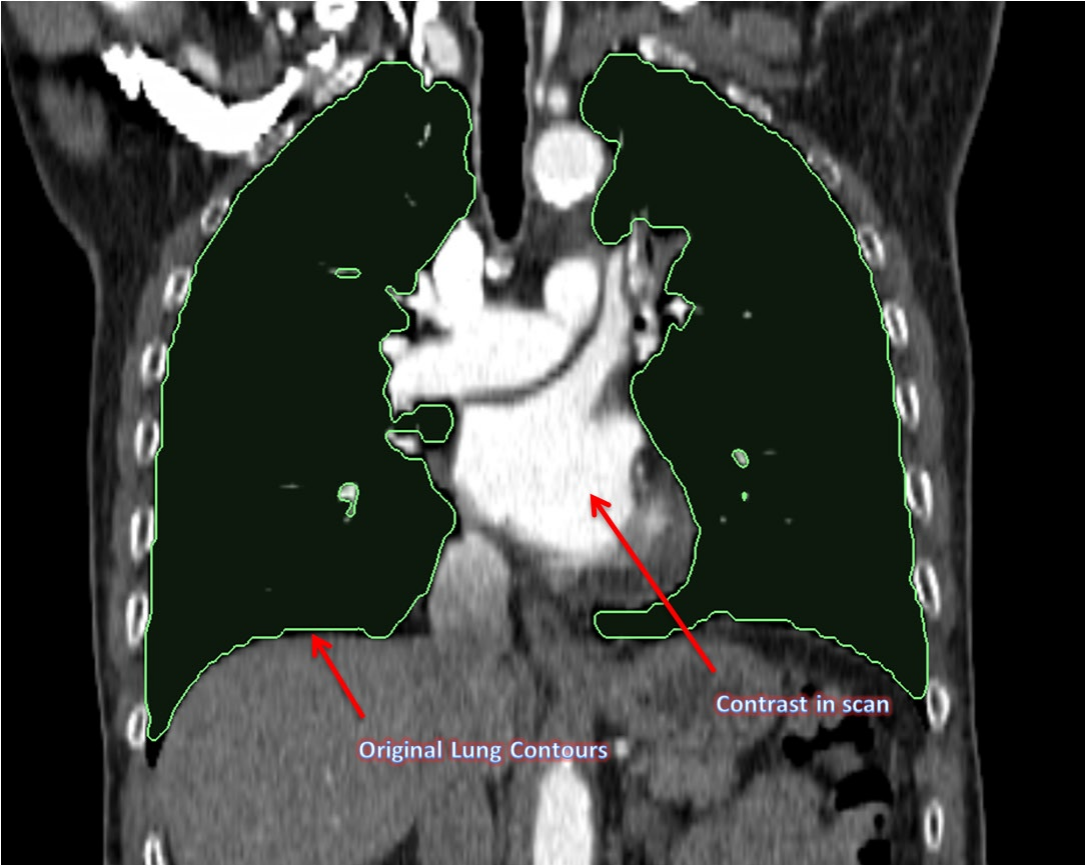
Coronal view of the original lung kvCT image showing the lung contours and contrast in scan.

**Figure 11:**
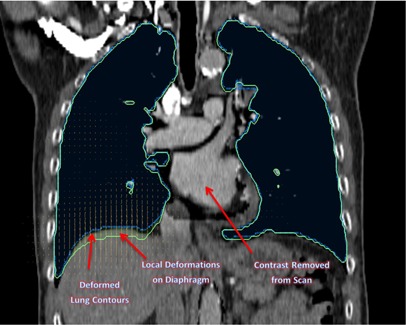
Coronal view of the same lung kvCT image from Fig. 10 illustrating the applied local deformations on the diaphragm and the changes in lung contour associated with that. This mimics an inhale and exhale breathing phase of respiratory cycle. The contrast is also taken out of this image to validate DIR during variable contrast enhancement.

Figures 10 and 11 show the coronal view of original kvCT image with contrast, and the coronal image from ImSimQA without contrast showing diaphragm motion and lung volume changes, respectively.

### C. Deformable image registration

The Insight Segmentation and Registration Toolkit (ITK) was used to perform (a) free‐form parametric deformable registration using a cubic B‐spline,^(^ ^54^ ^)^ and (b) nonparametric registration using diffeomorphic demons. The DIR and all the analysis tools described in this work were integrated into open source platform, 3D Slicer,^(^ ^55^ ^)^ via custom‐developed modules. We used the Mattes mutual information (MI) metric^(^ ^56^ ^)^ with an evolutionary algorithm followed by gradient descent optimizer for optimization.^(^ ^57^ ^)^ The images are initialized to line up their centers. The evolutionary optimizer works by searching for the minimum metric value by generating random samples about the current location in parametric space and iteratively growing or shrinking parameters of previous iteration to hone in on the optimum. This process is fairly resilient to noise. After this, a regular step gradient descent optimization is performed, with the transformation parameters incremented in the direction of gradient. The increment is determined in a bipartition manner until it converges on the minimum of the metric. Registration is performed in three phases: rigid, followed by affine, followed by deformable registration. A control point spacing of 60 (pixels), 50 maximum iterations, and 10% of the image pixels for metric evaluation, were used in this study during B‐spline registration to achieve optimal balance between quality of DIR and run time of registration. Registration was completed in 15 minutes on a Windows 7, 64 bit operating system running on Intel quad core 2.8 GHz processor with 8 GB RAM.

The same manually deformed images are also registered using diffeomorphic demons^(^ ^58^ ^)^ to provide a smooth and invertible transformation. In general, for nonparametric registration methods such as diffeomorphic demons used in this study, the registration is expressed as an objective function comprising of an image term and regularization term. The image term may be the difference in intensities of two voxels (optical flow as in demons), while the regularization term keeps the deformation field well‐behaved. This is usually done by smoothing the deformation field with a Gaussian at each iteration to ensure that it is well‐behaved.

Details on the implementation of this algorithm and its advantages over Thirion's demons algorithm^(^ ^14^ ^)^ are discussed in literature.^(^ ^58^ ^)^ A diffeomorphism by definition preserves the topology of objects in the image. In other words, it prevents folding of structures onto itself. Therefore the Jacobian is always nonnegative. This is a good property to have for medical image registration. The second important property of diffeomorphism is that it is guaranteed invertible by definition.

Registration is typically faster and takes about 10 minutes when using diffeomorphic demons algorithm using the same hardware platform as previously described.

### D. Evaluation scheme

In order for a thorough validation of DIR performance in a clinical environment, the following three characteristics were examined in this study.

#### D.1 Anatomical correspondence

Anatomical correspondence between original and deformed image sets can be identified using markers or contours defined by the users.

This validation is important because in radiotherapy clinical applications, the tumor and organs at risk (OAR) volume changes and, consequently, the partial volume dose received by these structures. The magnitude and location of these changes dictate the need for adaptive radiotherapy. Hence, the accuracy of DIR in relation to this is evaluated in this paper by quantitatively comparing the original tumor and OAR segmentation with those obtained from warping the RT structures with the deformation vector field (DVF) derived from registration. The ImSimQA DVF was used to warp the original RT structures in addition to CT images. The registration DVF from both algorithms was then applied to these RT structures. If the results of DIR were perfect, then the RT structures before and after DIR would be the same. The degree of mismatch indicates the quality of DIR from an anatomical correspondence perspective. Dice similarity coefficient, Hausdorff distance, and average surface distance were used as three metrics to evaluate the accuracy of tumor and OAR segmentation and spatial overlap index. These metrics have been previously used to compare segmentations in radiotherapy applications^(^ ^59^ ^–^ ^62^ ^)^ and are described below.

##### D.1.1 Dice similarity coefficient

The metric computes the number of pixels that overlap between the two volumes and normalizes it by the half the sum of the number of nonzero pixels in the two volumes. The result is a value between 0 (no overlap) and 1 (perfect overlap):

(1) $$\alpha =\frac{2X\left|A\cap B\right|}{\left|A\right|+\left|B\right|}$$

where *A* is the gold standard segmentation which in our case refers to segmentation in kvCT fixed image, and *B* is the segmentation mapped from the deformably registered image. The metric is symmetric and is sensitive to both differences in scale and position. While volume overlap is a good indicator of mismatch, it is a poor indicator of shape since is not a measure of distance and, hence, the following metrics are also evaluated to assess the overall accuracy.

##### D.1.2 Hausdorff distance

The Hausdorff distance^(^ ^63^ ^)^ is defined as the maximum of the closest distance between two volumes where the closest distance is computed for each vertex of the two volumes.

The metric is very sensitive to outliers since the most mismatched point is the sole determining criteria of the distance.

##### D.1.3 Average surface distance

This metric mitigates the outlier problem exhibited by the Hausdorff distance. The metric is the average of the absolute distance from each surface pixel in one image to its closest point on the other image.

#### D.2 Deformation field

The physical characteristics of the deformation fields should be investigated. This is because recent applications in adaptive radiation therapy (ART) have used the deformation fields arising from image registration process to warp the RT dose and display a deformed dose.^(^ ^64^ ^–^ ^67^ ^)^ Hence, some quantitative information on the physical characteristic of deformation fields is necessary for clinical implementation of ART. It is known that matching of structures based on their intensities alone is not a sufficient condition to produce physically achievable deformations.^(^ ^68^ ^)^

In this work we used a number of methods to evaluate the characteristics of the DVF. One of the key methods reported in the literature is the concept of inverse consistency.^(^ ^69^ ^–^ ^71^ ^)^ Inverse consistency between two images A and B are evaluated as follows in this paper. Image A is deformed to match image B, and image B is separately deformed to match image A using two different algorithms. A perfect inverse consistent algorithm will produce a true inverse DVF when the roles of source and target images are switched. However, in practice this is not the case. The inverse consistency error (ICE) between forward and inverse registration is calculated by compositive accumulation of forward and inverse deformation fields. The details of compositive accumulation are discussed elsewhere.^(^ ^69^ ^,^ ^72^ ^)^ If $D_{1}$ and $D_{2}$ are two deformation fields, a single warping by compositive addition of $D_{1}$ and $D_{2}$ is equivalent to successive deformation of an image by $D_{1}$ and then followed by $D_{2}$.

For the warp, we use a linear interpolator (i.e., we add the right field to the interpolated left field for that pixel as the resulting point x will not land exactly in the grid).

If the deformation maps are true inverses, this composition will yield zero. The L2 norm (absolute magnitude) of the composed fields is used to quantify the magnitude of inverse consistency error.

Further, ICE between the DVF arising from DIR and the synthetic DVF generated from ImSimQA software that was used to produce clinically relevant organ deformation was evaluated. The ImSimQA can also output inverse DVF of the applied deformation. This DVF was compared with the DVF generated from the inverse registration process where the roles of source and target images were switched. A compositive accumulation of the ImSimQA DVF and the DVF from registration (B‐spline and diffeomorphic demons) was done to quantify the ICE between DVFs. If the results of DIR produced a DVF which is the exact inverse of applied synthetic DVF in ImSimQA, then this composition of DVFs will be zero. The L2 norm of the composed DVFs is computed to quantify the ICE between DVFs.

Diffeomorphism is a necessary condition for deformation fields to be physically feasible.^(^ ^73^ ^)^ This property is related to the Jacobian of the deformation field. Negative Jacobians indicate unrealistic physically unachievable organ deformations, as organs can only be compressed and deformed but cannot undergo noninvertible spatial transformations like folding of structures.^(^ ^74^ ^,^ ^75^ ^)^ This is the primary advantage of diffeomorphic demons over the B‐spline algorithm, as the Jacobian is always nonnegative in the former.

In this study, we computed the determinant of the Jacobian of the deformation field as a criterion for validating physical behavior of deformation. The Jacobian of the deformation field gives information about the image transformation consistency.^(^ ^75^ ^,^ ^76^ ^)^ A determinant greater than 1 indicates expansion at that location, while a value less than 1 indicates contractions.

The harmonic energy of the deformation field arising from B‐spline and diffeomorphic demons algorithms was calculated in this study to quantify the regularity of the spatial transformation obtained by the deformable registration process.^(^ ^77^ ^)^ The harmonic energy captures the nonlinearity of the warp (i.e., deviation from an affine transformation) and is inversely proportional to smoothness of the deformation field.^(^ ^77^ ^)^

#### D.3 Image characteristics

Comparison between original and deformably registered images to provide a measure of how well the deformation is recovered in the entire image voxel space.

We used mean squared error (MSE) as the metric to define the extent of mismatch between the original image A and the deformably registered image B, which is the normalized square difference between the two images A and B.^(^ ^78^ ^)^ For a perfect image match between images A and B, the MSE error is zero. The error is reported as Root MSE in the Results section below, where Root $\text{MSE}=\sqrt{\text{MSE}}$

Three clinically relevant examples from prostate, head and neck, and lung cases are presented, and the accuracy of DIR is evaluated using various methods described above and the relative merits of these are discussed.

## III. RESULtS

The results of the accuracy of DIR evaluation in three anatomical sites namely prostate, head and neck, and lung are presented below

### A. Inverse consistency error

**Figure 12:**
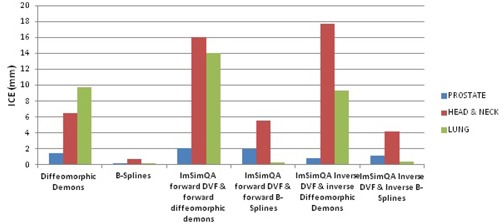
Inverse consistency error comparison.

Figure 12 lists inverse consistency error (ICE) between various DVFs used in DIR and ICE between applied ImSimQA DVF and DVF from DIR for the three anatomical sites studied.

**Figure 13:**
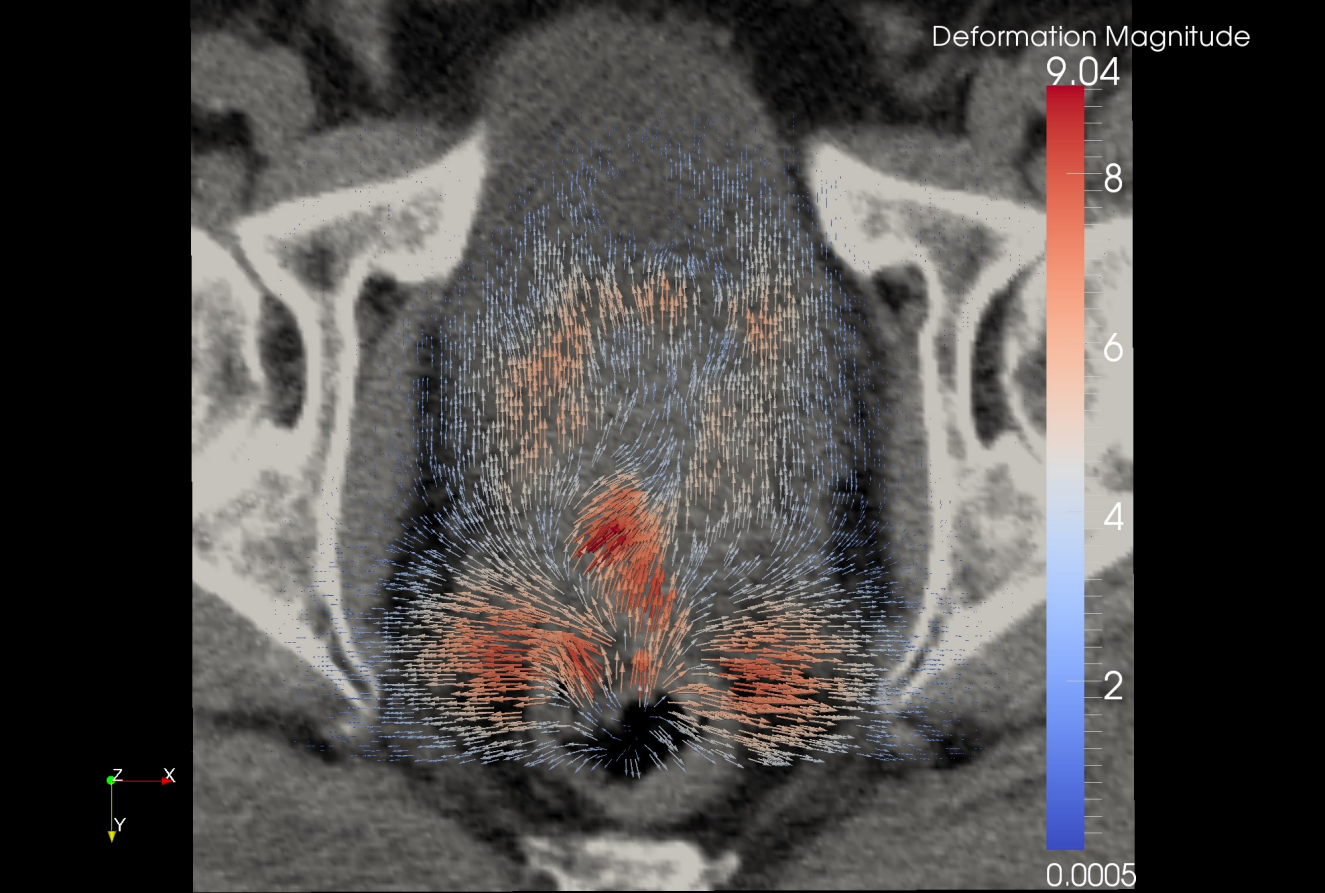
Forward diffeomorphic demons DVF from the registration overlaid on the original prostate kvCT image illustrating the local changes due to the DVF. The field vectors are pointing outward.

**Figure 14:**
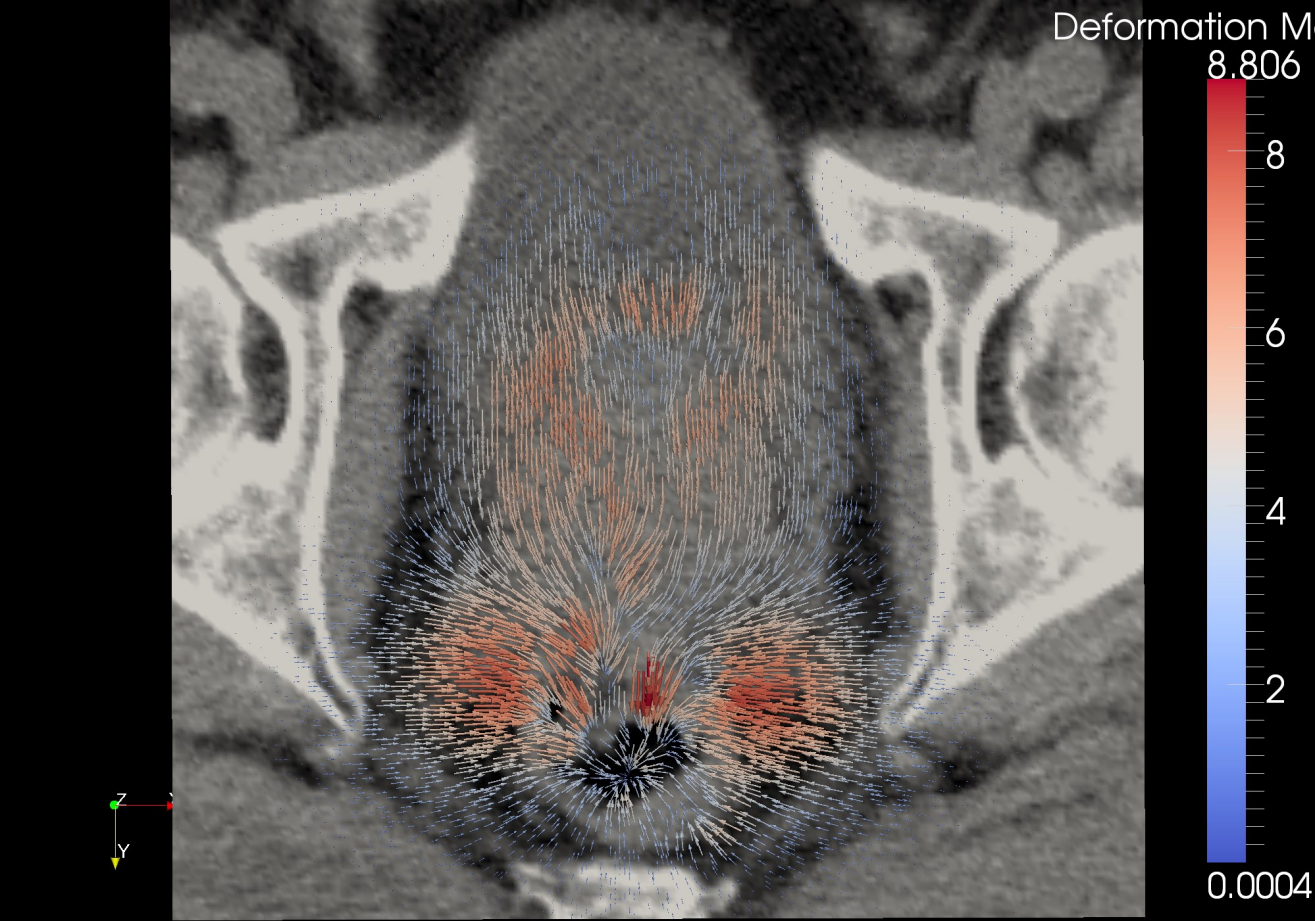
Inverse diffeomorphic demons DVF when the roles of source and target images were switched from previous example, overlaid on the original kvCT image. The field vectors are pointing inward.

**Figure 15:**
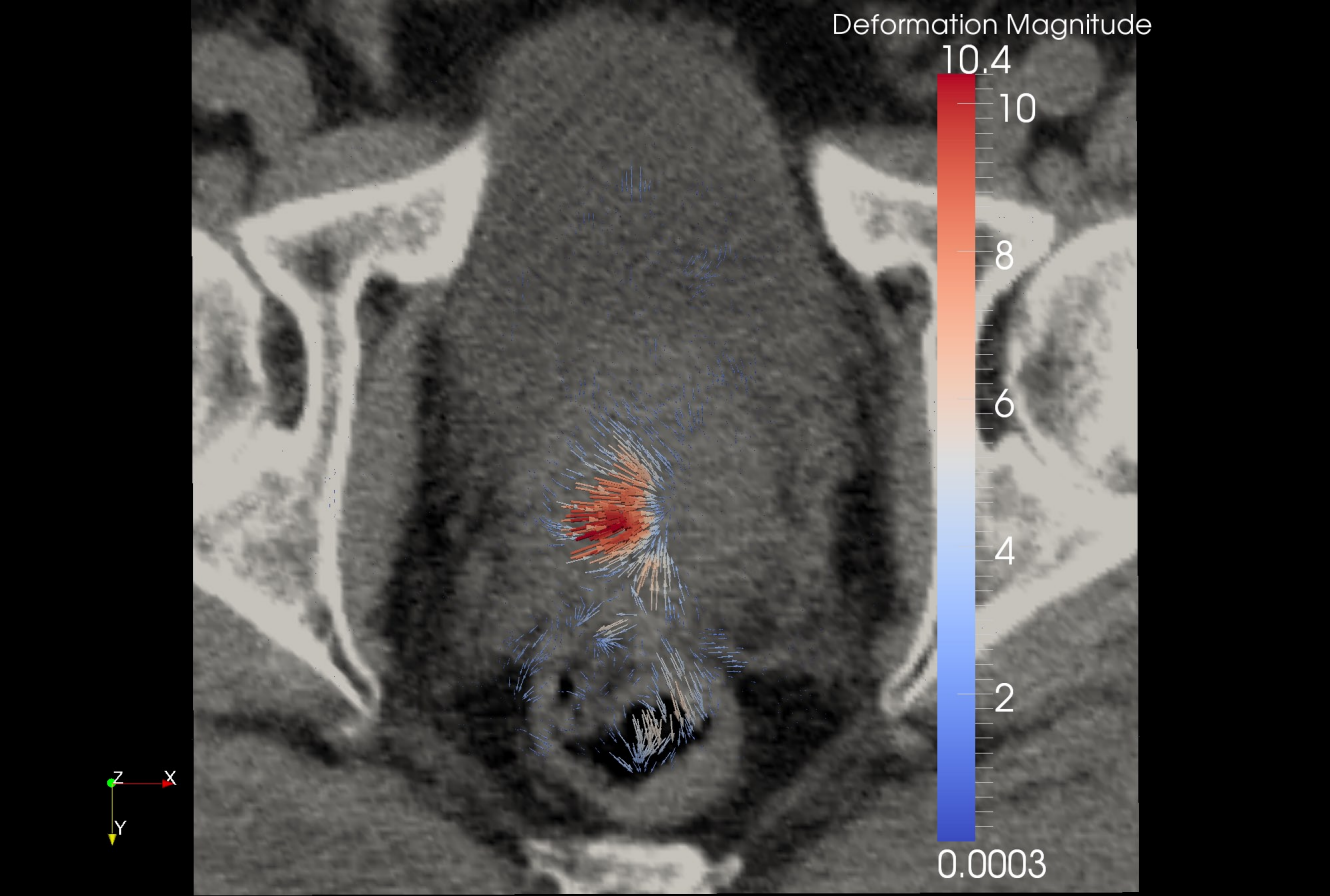
Compositive addition of forward and inverse demons DVF overlaid on the original kvCT image. If the algorithm were truly inverse‐consistent, this composition would yield zero. The magnitude of this compositive addition is 1.45 mm in this example (as discussed in Figure 12).

As an example for the prostate case, the DVF from diffeomorphic demons algorithm is overlaid on the original kvCT image for forward, inverse, and compositive addition of forward and inverse DVFs is shown in Figs. 13, 14, and 15, respectively. Figure 15 relates to the quantitative ICE described above (1.45 mm) for diffeomorphic demons algorithm for the prostate case.

### B. RMSE, Jacobian, and harmonic energy of DVF

Table 1 lists root mean square error, minimum Jacobian, and harmonic energy of deformation field for registration algorithms both in forward and inverse directions for all three anatomical sites. The MSE and (hence) RMSE in lung case for diffeomorphic demons algorithm is large due to the improper estimation of displacement fields arising due to variable contrast enhancement and, hence, Jacobian is not calculated for demons algorithm.

**Table 1:** RMSE, Jacobian, and harmonic energy of DVF.

	*Prostate*				*Head & Neck*				*Lung*			
*Algorithm*	*RMSE Before DIR*	*RMSE After DIR*	*Harmonic Energy*	*Minimum Jacobian*	*RMSE Before DIR*	*RMSE After DIR*	*Harmonic Energy*	*Minimum Jacobian*	*RMSE Before DIR*	*RMSE After DIR*	*Harmonic Energy*	*Minimum Jacobian*
Diffeomorphic Demons Forward	25.99	11.82	0.05	0.12	187	51.9	0.32	0.0005	91.33	853.6	0.53	N/A
Diffeomorphic Demons Inverse	25.99	11.64	0.09	0.003	187	66.1	0.43	0.003	91.33	223.3	0.19	N/A
B‐spline Forward	25.99	11.03	0.0006	0.88	187	80.8	0.014	0.53	91.33	68.1	0.0005	0.82
B‐spline Inverse	25.99	10.9	0.0005	0.87	187	51.2	0.005	0.58	91.33	69.2	0.0004	0.87

### C. Accuracy of RT structures

Table 2 evaluates the accuracy of RT structures for the prostate case, after DIR, when compared to original segmentation done by the radiation oncologist in kvCT (used as the gold standard) for both diffeomorphic demons and B‐spline algorithms. This was done by applying the registration DVF to RT structures deformed by ImSimQA DVF. All the evaluation is done on the original fixed image (kvCT) coordinate system.

**Table 2:** Accuracy of RT structures after DIR (prostate).

Algorithm: Diffeomorphic Demons			
*Anatomy*	*Dice Similarity Coefficient*	*Hausdorff Distance (mm)*	*Average Surface Distance (mm)*
Prostate	0.85	15.9	2.3
Bladder	0.93	11.1	0.78
Rectum	0.79	12.6	1.2
Femoral Heads	0.99	1.7	0.1
Mean values	0.89	10.3	1.1

Algorithm: B‐spline			
*Anatomy*	*Dice Similarity Coefficient*	*Hausdorff Distance (mm)*	*Average Su Distance (mm)*
Prostate	0.91	9.8	1.03
Bladder	0.95	7.7	0.42
Rectum	0.89	10.3	0.8
Femoral Heads	0.99	1.2	0.1
Mean values	0.94	7.3	0.59

Table 3 evaluates the accuracy of RT structures for head & neck case. Although by visual inspection of the images the registration seems to agree qualitatively (the skull and vertebral bodies matched after DIR), the contour comparison statistics are not clinically acceptable, especially for organ‐at‐risk structures. This is primarily due to large neck flexion introduced as a known deformation in ImSimQA. Based on this analysis, auto‐registration of images when there is significant neck flexion should be evaluated with caution, especially when there is a retreatment being considered.

**Table 3:** Accuracy of RT structures after DIR (head and neck).

Algorithm: Diffeomorphic Demons			
*Anatomy*	*Dice Similarity Coefficient*	*Hausdorff Distance (mm)*	*Average Surface Distance (mm)*
PTV Primary	0.85	8.9	1.8
PTV Secondary	0.86	9.1	1.5
Spinal Cord	0.51	12.2	2.4
Right Parotid	0.84	4.7	0.8
Left Parotid	0.77	6.6	1.4
Brainstem	0.64	11.9	2.7
Mandible	0.63	40.5	4.6
Larynx	0.86	5.7	1.1
Right Eye	0.74	7.5	1.8
Left Eye	0.79	4.9	1.3
Mean values	0.75	11.2	1.9

Algorithm: B‐spline			
*Anatomy*	*Dice Similarity Coefficient*	*Hausdorff Distance (mm)*	*Average Surface Distance (mm)*
PTV Primary	0.88	8.2	1.5
PTV Secondary	0.87	8.6	1.4
Spinal Cord	0.52	10.5	2.2
Right Parotid	0.84	3.7	0.8
Left Parotid	0.79	5.9	1.3
Brainstem	0.52	9.7	3.9
Mandible	0.7	13.6	1.9
Larynx	0.86	5.7	1.1
Right Eye	0.59	8.7	2.6
Left Eye	0.83	4.7	0.93
Mean values	0.74	7.9	1.7

Table 4 computes the accuracy of RT structures for the lung example involving variable contrast enhancement. The diffeomorphic demons algorithm produced improper displacement estimation in this case because of the difference in intensities of two images due to the variable contrast enhancement. The mismatch in RT structures is particularly relevant in heart, lung, bronchial tree, and vertebral bodies as the Hausdorff distance exceeds 10 mm and the average surface distance is as large as 11.8 mm for heart. This is because diffeomorphic demons algorithm tries to match structures of same intensity which, in our case, does not correspond to identical anatomical structures due to the differences in contrast between two images.

**Table 4:** Accuracy of RT structures after DIR (lung).

Algorithm: Diffeomorphic Demons			
*Anatomy*	*Dice Similarity Coefficient*	*Hausdorff Distance (mm)*	*Average Surface Distance (mm)*
PTV	0.83	8.8	1.4
Cord	0.95	4.6	0.2
Heart	0.37	56.7	11.8
Lung	0.99	17.7	0.4
Bronchial Tree	0.49	18	4.2
Trachea	0.91	15	1.3
Vertebral Body	0.92	10.3	0.2
Mean Value	0.78	19.3	2.8

Algorithm: B‐spline			
*Anatomy*	*Dice Similarity Coefficient*	*Hausdorff Distance (mm)*	*Average Surface Distance (mm)*
PTV	0.88	6.9	2.1
Cord	0.94	8.3	0.3
Heart	0.99	4.1	0.2
Lung	0.99	6.3	0.3
Bronchial Tree	0.96	1.4	0.1
Trachea	0.97	1.4	0.2
Vertebral Body	0.93	10.3	0.2
Mean Value	0.95	5.5	0.4

## IV. DISCUSSION

Deformable image registration will continue to be a key component in the implementation of adaptive radiotherapy with the ultimate goal of dose tracking and dose accumulation based on daily image feedback.^(^ ^41^ ^,^ ^66^ ^,^ ^67^ ^)^ Verification of DIR accuracy is an important task in implementation of adaptive radiotherapy. We have presented a framework to test and evaluate the accuracy of DIR using known deformations, which are clinically relevant, that can be applied to any CT images. The accuracy of DIR was evaluated by comparing anatomical correspondence, physical characteristics of deformation field, and image characteristics. The relative merits of these methods in the final decision‐making on DIR accuracy for the anatomical sites studied is discussed below.

***Prostate:*** Our results on prostate case indicate that the ICE was comparable to both algorithms. Also, the MSE values were very similar for both methods. However the B‐spline algorithm had significantly better anatomical correspondence for rectum and prostate than the diffeomorphic demons algorithm. So, considering the anatomical correspondence of the RT structures, one can conclude that the B‐spline algorithm performed better. In this example, the MSE and ICE evaluation parameters provide no criteria to determine which method performs better.

***Head and Neck:*** For the head and neck case, the ICE was much larger for the demons algorithm (6.5 mm) as compared to B‐spline (0.7 mm). The MSE was comparable for both algorithms. However, since the induced neck flexion was large, neither algorithm had a desired anatomical correspondence for PTV and organs at risk that could make the result clinically acceptable. Similar to the prostate case, this example also indicates that considering only the ICE and MSE methods could lead to false positive conclusions.

***Lung:*** In the lung case, the B‐spline algorithm accurately estimated the deformations between images with variable contrast and was clearly superior in all the metrics that were evaluated. The demons algorithm had gross errors in areas of contrast differences between images. This was the only example where all metrics used for the DIR evaluation were in full agreement on the decision‐making of the DIR algorithm performance.

Verification of absolute accuracy of DIR is a challenging problem, as each of the methods studied has its own drawback. In the case of inverse consistency, a zero value for ICE is a necessary, but not sufficient, condition for an accurate algorithm as errors in one DVF may cancel out errors in the other to yield a net zero value during composition of two deformation maps.

The determinant of the Jacobian and the harmonic energy of the deformation field was used to classify the registration strategies based on invertibility and smoothness, although it does not give information on the accuracy of DIR. However, one needs to confirm the nonnegative value of Jacobian of the deformation field to ensure that a given DVF is physically achievable by an organ.^(^ ^72^ ^,^ ^74^ ^,^ ^75^ ^)^ The harmonic energy captures the nonlinearity of the warp.

The harmonic energy of B‐spline was consistently lower in all our examples, and was generally small since it was physically constrained. The parameters that control it are the maximum deviation the user allows (step length) during registration and the number of nodes specified in the command line. The harmonic energy of the diffeomorphic demons is controlled by the parameter “sigma” used to smooth the deformation field. Increasing sigma will reduce the harmonic energy, but will come at the expense of reduced registration accuracy. The harmonic energy from B‐spline registration was consistently lower on all our cases, indicating that the deformation field from B‐spline was smoother. An abnormally large value of harmonic energy may indicate problems with DVF, as was the case for demons algorithm during registration of images with variable contrast enhancement. However, there is nothing in the B‐spline algorithm that prevents negative Jacobians, which is physically unrealistic. Deformation fields from diffeomorphic demons, on the other hand, are guaranteed invertible and the Jacobian is always nonnegative.

The image quality of a deformed image set and product of a DIR method is significant for the daily clinical routine when used to define OARs and target volumes. However, the use of the MSE as image quality metric is proven to be inadequate for drawing a useful and consistent conclusion. A small value of MSE indicates an overall good accuracy in the entire image voxel space, but does not guarantee good accuracy of DVF inside the organs. Another option to address this issue is to make a selective MSE calculation within regions of interest (e.g., OARs) and investigating other image quality metrics. If unsure about the DIR image outcome, the images should be reviewed by a clinical expert.

Ultimately the accuracy of DIR also needs to be validated with contour comparison methods, as outlined in this study, because the registration accuracy of RT structures and, hence, the partial volume doses received by these structures dictate the need for adaptive radiotherapy. This evaluation proved to be the most consistent and reliable method in validating DIR accuracy in our study.

DIR results in a daily clinical environment might be very variable and affected by various factors such as patient anatomy, image quality, and registration parameters of the particular algorithm. It should be well‐appreciated that the evaluation of a DIR algorithm for use in a clinical routine should be conducted in a long‐term study that includes a large number of clinical cases.

## V. CONCLUSIONS

We conclude that the proposed framework offers the application of known deformations on any patient or phantom image sets that provide clinical medical physicist tools to test, understand, and quantify limitations of each algorithm before implementing deformable image registration in the clinic. The evaluation based on anatomical correspondence, physical characteristics of deformation field, and image characteristics can facilitate DIR verification with the ultimate goal of implementing adaptive radiotherapy. The suitability of application of a particular evaluation metric in validating DIR is dependent on the clinical deformation observed.

## ACKNOWLEDGMENTS

This work was partially supported by the National Institute of Health grants (1R01CA154491‐01).
